# Assessment of the magnitude, economic impact, and factors associated with expired veterinary pharmaceuticals in animal health facilities in South Wollo, Ethiopia

**DOI:** 10.3389/fvets.2024.1390891

**Published:** 2025-01-07

**Authors:** Yesuneh Tefera Mekasha, Ermias Belay Mekonnen, Abebe Tesfaye Gessese, Kassahun Berrie, Achenef Melaku Beyene, Bereket Dessalegn

**Affiliations:** ^1^Pharmaceutical Sciences, Pharmaceutical Quality Assurance and Regulatory Affairs, University of Gondar, Gondar, Ethiopia; ^2^Department of Veterinary Medicines, College of Veterinary Medicine and Animal Sciences, University of Gondar, Gondar, Ethiopia; ^3^Department of Veterinary Biomedical Sciences, College of Veterinary Medicine and Animal Sciences, University of Gondar, Gondar, Ethiopia; ^4^Veterinary Epidemiology and Public Health, College of Veterinary Medicine and Animal Sciences, University of Gondar, Gondar, Ethiopia; ^5^Department of Medical Microbiology, College of Medicine and Health Sciences, University of Gondar, Gondar, Ethiopia; ^6^Department of Veterinary Pathobiology, College of Veterinary Medicine and Animal Sciences, University of Gondar, Gondar, Ethiopia

**Keywords:** cross-sectional study, magnitude, financial impact, associated factor, animal health facility, Dessie town, South Wollo, Ethiopia

## Abstract

**Background:**

The issue of veterinary pharmaceutical expiration is a significant concern in animal health facilities globally. The existence of veterinary pharmaceutical expiration can be mainly associated with inadequate inventory control, store management, and a lack of effective pharmaceutical regulatory policies and guidelines. Hence, the study aimed to evaluate expired veterinary pharmaceuticals’ scope, economic impact, and contributing factors.

**Methods:**

A cross-sectional, explanatory sequential study design involving a mixed quantitative and qualitative approach was employed among 13 animal health facilities from March 2022 to December 2023 in and around Dessie town, South Wollo, Ethiopia. A key informant interview guide was used to retrieve the qualitative data that were analyzed through thematic content analysis. Then, the collected data were coded and analyzed using SPSS version 25. A mean score was used to determine the critical factors associated with veterinary pharmaceutical expiration.

**Results:**

The study found that the magnitude of expired veterinary pharmaceuticals in the fourth fiscal year was 7%. This wastage rate of veterinary pharmaceuticals led to a loss of approximately 69,564.54 USD. From expired veterinary pharmaceutical unit pack perspectives, approximately 403-unit packs (66%) expired in veterinary clinics, resulting in a loss of approximately 38,229.33 USD, and 209-unit packs (34%) expired in private veterinary pharmacies, incurring a loss of approximately 31,335.22 USD. From Anatomical Therapeutic Classification (ATC), antibiotics accounted for 14.8% of the total financial loss. Additionally, approximately 53% of liquid dosage forms were expired. The quantitative study identifies the lack of an information system and necessary software, poor store management, and lack of strict accountability as critical contributors to veterinary pharmaceutical expiration. Additionally, inadequate inventory management systems and a lack of adherence to established policies and guidelines for managing veterinary pharmaceutical expiration were the most vital contributors as key informants cited.

**Conclusion:**

The financial burden associated with expired veterinary pharmaceuticals exceeded the permissible threshold of 2%, indicating a significant concern for animal health budgets and the aquatic environment. This study underlines that the issue of veterinary pharmaceutical expiration is a critical problem that necessitates policy implications. To mitigate the expiration rate of veterinary pharmaceuticals, collaboration among multidisciplinary veterinary professionals, the Ethiopian Agricultural Authority, pharmaceutical supply chain agency, and researchers is essential.

## Introduction

Ethiopia has a massive livestock population and is ranked among the top 10 countries in the world and the first country in Africa with an estimated population of 70 million cattle, 42.9 million goats, and 52.5 million sheep (1). However, the country struggles with the effective utilization of veterinary pharmaceutical products due to a weak legislative framework, inadequate veterinary practices, a lack of essential equipment and supplies, and failure to implement pertinent veterinary drug policies and guidelines (2–4). Additionally, the absence of domestic manufacturing companies in Africa, particularly Ethiopia, was yet another obstacle that led to the importation of foreign veterinary products into the pharmaceutical market of the country (5). The health budget of Ethiopia increased due to the increased importation of veterinary products, with pharmaceuticals worth 812 million US dollars in 2021, compared to 691 million US dollars in 2018 (6).

Moreover, the animal health facility currently requires strong regulatory oversight (7). Failure to follow an appropriate dispensing practice guideline within an animal health facility leads to a substantial amount of pharmacologically critical medications reaching their expiration date. This, in turn, leads to an adverse effect on the financial burden of the country as it incurs unnecessary expenses (8). Monitoring the animal health facility is critical to combating the wastage of veterinary pharmaceuticals. As the expired medication leads to monetary losses and makes it difficult for diseases in animals to obtain essential pharmaceuticals, it can lead to antimicrobial resistance, negatively impacting aquatic environments (9). Additionally, the expired drugs when disposed of improperly enter into the environment, particularly in the water bodies, can disrupt the food chain and affect aquatic species such as fish, invertebrates, and other microorganisms (10).

Based on available data, approximately 22.6% of veterinary medication outlets in Central Uganda had veterinary pharmaceuticals that had expired, and the majority of them had stock worth less than $200 million. The budget allotted for the protection of animal health throughout Africa has been challenged by the presence of expired veterinary medications in animal health institutions (8). Aside from their financial impact, expired medications are contested as one of the health strategies for a successful method of resolving complicated medical issues involving several disciplines, representing an integrated view of health made up of three interconnected domains: environmental, animal, and human (11).

The literature pointed out that, especially in Ethiopia, the procedure for disposing of outdated veterinary medications has not yet been put into place. The animal health facility disposal system is immature as compared to the human pharmaceutical preparation disposal system (2). The practice of disposing of expired veterinary products in developed countries is good when compared to sub-Saharan African continents. A witness study conducted in southern Brazil revealed that burning was the most common measurement tool in the disposal of expired veterinary drugs, leftovers, and wrappers (12).

The challenge of expired veterinary drugs staying within the supply chain or posing animal and public health risks is of global concern, especially in low-income countries, including Ethiopia, where a lot of veterinary medicines are expired due to negligence and poor financial systems in animal health facilities. The use of ineffective, poor-quality, and harmful veterinary drugs can result in therapeutic failure, exacerbation of the disease, resistance development to drugs, and sometimes death (13). There is a significant need to address the issue of waste disposal systems across all animal health facility environments. While the amount and environmental impact of human healthcare waste are well quantified, considerably less is known about the amount and type of waste explicitly generated in the delivery of veterinary care. Addressing waste is a top priority for veterinary staff and students, who generally seek to elevate sustainability across the profession (14). In 2020, veterinarian Sustain an association of veterinary professionals in the United Kingdom proposed “a no-waste society” as one of six veterinary sustainability goals whereby they “minimize the usage and disposal of resources and materials and support a transition to a circular economy (15). Additionally, the ChemClear^®^ program in Australia is a national program that aims to safely dispose of veterinary medications that have expired. This is an important step toward responsible chemical management and environmental protection in the country (9).

Unfortunately, addressing the disposal issue of waste veterinary medicines across the professional level is extensive work, especially in Ethiopia, where the veterinary supply chain agency gives little attention to expired veterinary pharmaceuticals (2). Furthermore, the regulatory framework in Ethiopia for veterinary drugs lacks effectiveness in overseeing antimicrobial stewardship and post-market surveillance due to inadequate collaboration between the Ethiopian Agricultural Authority and veterinary drug experts (16). Consequently, significant quantities of medications have reached their expiration dates within the animal health facility. A study from the northern part of Ethiopia revealed that a poor regulatory level of coordination was detected (85.4%) between the regulatory body and veterinary drug professionals (16). This could lead to the discharge of obsolete veterinary medications into the environment without considering the health of the broader public due to a lack of regulatory enforcement regarding the penalties for professionals involved in the drug trade.

It is critical to assess the factors associated with the expiration of pharmaceuticals in animal health facilities, aside from considering the volume and monetary loss of expired drugs. This evaluation is essential in order to establish veterinary pharmaceutical policy and implementation of standards that align with veterinary treatment guidelines for the appropriate use of these medications (8). In human medicine supply chains, medication expiries are brought on by a lack of stock monitoring, a lack of knowledge about preventive measures, a lack of action on the part of physicians in quantifying medications, profit-driven quantification, excessive stockpiling, and vertical procurement (17). However, in Ethiopia, inadequate data exist on managing expired veterinary medicines in animal health facilities, with factors such as poor storage conditions, improper handling, and improper disposal being significant obstacles (2). The study indicated that 37 veterinary health facilities in four zones, namely, South Gondar, West Gondar, Central Gondar, and West Gojjam zones and Bahir Dar administrative city found that 59.5% of them had inadequate stock management practices (2).

As the evidence showed, the extent, financial impact, and causes of expired veterinary medications in Dessie town and south Wollo areas of Ethiopia are lacking (18). The research confirms that the discharge of expired veterinary medications into the surroundings may result in the development of resistance to antimicrobial treatments (19). The report showed that out of 384 samples examined in Wollo districts, carcass swabs (79.6%) and milk tank samples (16.7%) showed the highest *Escherichia coli* prevalence (20). The main contributors to the antibiotic-related catastrophe are livestock products containing antibiotic residues, environmental discharge, improper use of antibiotics, and insufficient oversight of drug manufacturing, usage, and disposal (21). Additionally, industrialization has led to increased antibiotic residues in food and environment, causing the development and spread of antibiotic-resistant bacteria and their resistance genes (22).

The existing evidence revealed that in order to formulate policies to reduce losses and maintain therapeutically critical pharmaceuticals, inspecting the veterinary pharmaceutical circumstances in Ethiopia with an emphasis on magnitude, financial implications, and factors impacting expiration was critical (23, 24). Taking the aforementioned issues into consideration, the study aimed to assess the magnitude, associated factors, and disposal system of expired veterinary pharmaceuticals within animal health facilities.

## Materials and methods

### Study setting

**Figure 1:**
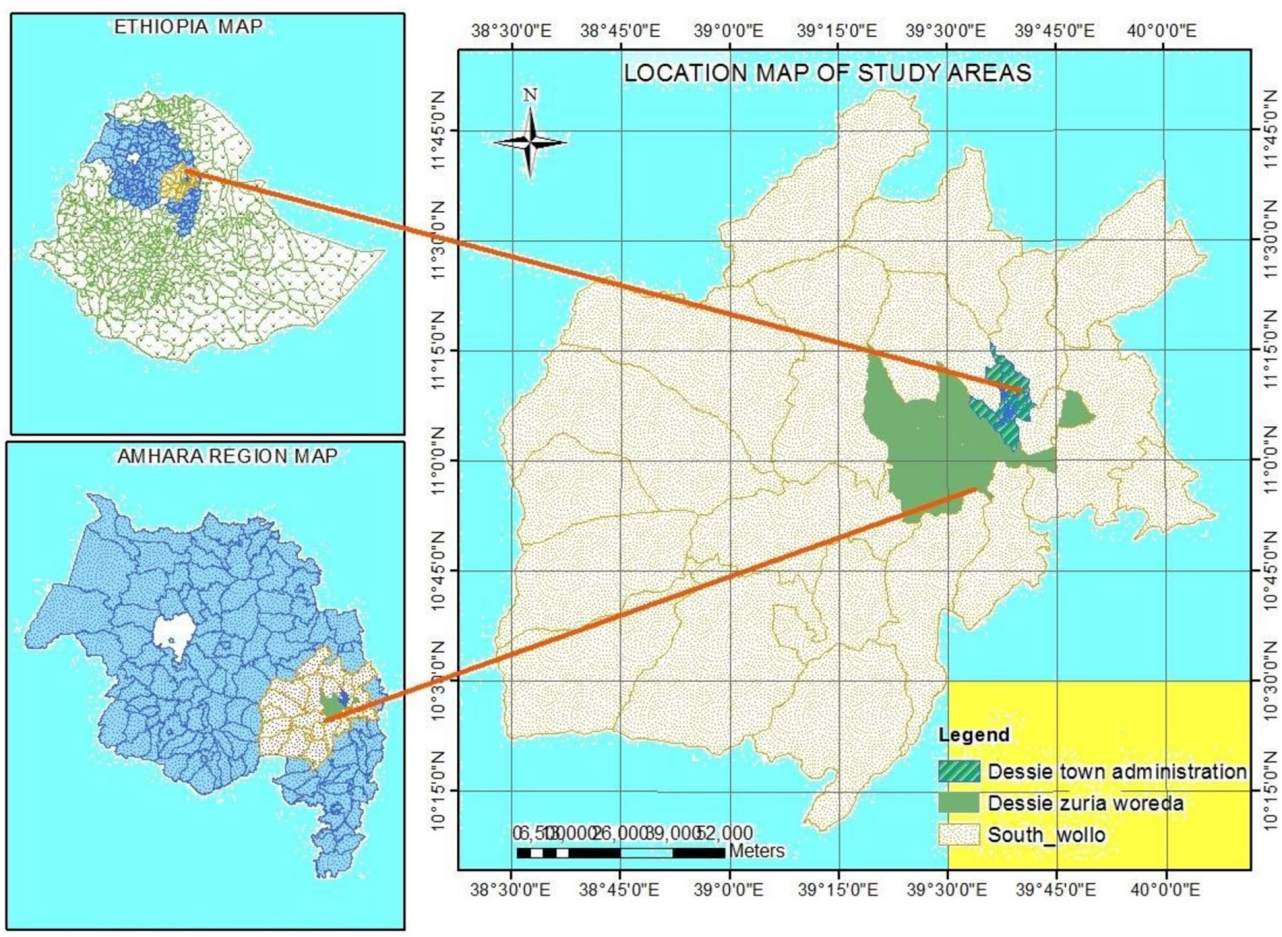
Map of the study area (51).

The study was conducted in and around Dessie, South Wollo, Amhara Region, Ethiopia, from March 2022 to December 2023 (Figure 1). An extensive report by the International Livestock Research Institute (ILRI) revealed that the advancement of animal health services in the vicinity of Dessie town is being actively pursued. This progress is primarily attributed to the active participation of public veterinary clinics and animal health posts (*n* = 6), private veterinary clinics (*n* = 4), and private veterinary pharmacies (*n* = 4) (25). The major livestock species found in the areas are cattle, goats, equines, and sheep. The livestock population in the district of the study area is also estimated to be 99,128 cattle, 177, 324 sheep, 38, 055 goats, 31,240 equines, 116, 712 poultry, and 6,445 bee colonies (26).

### Study design and research questions

A cross-sectional study with an explanatory sequential study design was conducted in and around Dessie town South Wollo Ethiopia within animal health institutions. Quantitative data were first gathered and examined. Following that, qualitative data were collected and inspected in light of the quantitative conclusions. In order to collect pertinent data for both quantitative and qualitative analyses of expired veterinary medications, interviews and retrospective data evaluation were employed.

To determine the magnitude and monetary loss resulting from expired veterinary pharmaceuticals in the selected location, the collected data on expired veterinary pharmaceuticals were quantitatively described. The lead investigator conducted in-depth face-to-face interviews with veterinarian clinicians, veterinary clinics, drugstores, and animal science professionals in order to perform a qualitative study. These interviews served to gain a thorough understanding of the situation of expired veterinary pharmaceuticals, pinpoint the causes of their expiry, and investigate feasible preventative measures to reduce the incidence of expired veterinary medications. The study objective was achieved using five research questions, such as (1) What amount of veterinary pharmaceuticals that has expired is there in and near Dessie’s animal health facilities? (2) Which veterinary pharmaceutical categories are most frequently discovered to be expired at these facilities? (3) How do the animal health facilities handle and get rid of expired veterinary pharmaceuticals? (4) What are the main causes of the expired veterinary pharmaceuticals found in these animal health facilities? (5) What strategies or preventative measures are being taken in these facilities to reduce the likelihood that veterinary pharmaceuticals would expire?

### Study population and source

All animal health facilities found in and around Dessie, South Wollo, Ethiopia, were considered under source of population. Governmental veterinary pharmacies, governmental veterinary clinics, private veterinary pharmacies, and private veterinary clinics that record books of expired veterinary drugs and all inventory management records of the past four fiscal years (2019, 2020, 2021, and 2023) in all animal health facilities that fulfill inclusion criteria were considered as the study population.

### Sampling techniques

A total of 13 animal health facilities were selected for this study, consisting of five veterinary clinics, and eight private veterinary pharmacies. These facilities were selected in and around Dessie town using a census-based sampling technique as they offer a wide range of veterinary services to the community.

The self-administered structured questionnaire was distributed to various study participants, including the veterinary clinician from the district veterinary clinic, the manager of the district veterinary clinic drug store, and an animal science expert. These participants were requested to complete the questionnaire, which consisted of a checklist for evaluating animal health facilities. The questionnaire aimed to assess the extent of expired veterinary pharmaceuticals (Supplementary File 1), the proper handling of expired veterinary pharmaceuticals (Supplementary File 2), and the factors contributing to the presence of expired veterinary pharmaceuticals (Supplementary File 3). From the 13 animal health facilities, a total of 20 study participants were selected purposively to ensure information-rich data. A purposive sampling method was employed to select veterinary clinicians (*n* = 9), store managers (*n* = 8), and animal science experts (*n* = 3) at each animal health facility, as they were considered to possess more knowledge than other health professionals. The determination of the extent and monetary value was based on expired veterinary pharmaceuticals with recorded prices from the fiscal years 2019/2020 and 2021–2023 G.C.

For the qualitative study, the veterinary clinician, store manager, and animal science expert in the selected facilities were purposefully selected. The number of key informants was determined depending on the saturation of information concerning emerging themes. Interviews were conducted with key informants within the facilities to explore the factors responsible for the expiry of veterinary pharmaceuticals and possible intervention mechanisms to reduce the extent of the expiration rate in the animal health facility.

### Inclusion and exclusion criteria

#### Inclusion criteria

All governmental and private veterinary pharmacies and clinics (*n* = 13) found in and around Dessie town that are active and fulfill the Veterinary Drugs and Animal Feed Administration and the Control Authority of Ethiopia were included in the study (27). Additionally, veterinary clinics and pharmacies with records of expired veterinary drugs and having data on expired veterinary drugs for at least 4 years prior to the collecting period were included.

All the study participants who were present at the study site and volunteered to participate in the study during the study period were included as were all expired veterinary drugs recorded with prices in the previous four fiscal years (2019, 2020, 2021, and 2023 years).

#### Exclusion criteria

The study excluded veterinary professionals who did not volunteer to participate in data collection, and veterinary clinics and pharmacies provided inadequate data records. The North Ethiopian War in 2022 G.C resulted in significant destruction of data and documents, leading to fragmented recorded files on expired veterinary pharmaceuticals in the targeted animal health facilities. Additionally, many private animal health facilities had disorganized documents concerning expired veterinary pharmaceuticals.

### Study variables

#### Independent variables

Facility demographic variables such as animal health facility level, year of established, additional budget of facility, inventory management system, document, and record: standard operating procedure, and its implementation (stock card, bin card, standard treatment guideline, essential drug list), mostly expired veterinary pharmaceuticals, and personnel demographic variables such as age, gender, marital status, profession, work experience, and educational level were included as independent variables.

#### Dependent variables

The magnitude of expired veterinary pharmaceuticals and the level of veterinary pharmaceutical expiration (high and low) could be included as dependent variables.

### Data collection tools and techniques

Previously published literature and guidelines were used for the quantitative study to review all records of the expired veterinary drug file and to abstract secondary data on the extent, and types of expired veterinary pharmaceuticals, monetary loss, and disposal practice. Expired veterinary drugs found at each facility were studied for their type, unit, dosage forms, cost, and quantity using the adopted format from the Ethiopian Veterinary Drug and Feed Administration and Control Authority (27) and published literature in peer-reviewed journals (28, 29).

The 5-point Likert scale was employed for assessing factors associated with the expiration of veterinary drugs in the studied animal health facilities. The facility was assessed using a Likert scale that ranged from strongly disagree (*n* = 1) to strongly agree (*n* = 5).

A self-administered questionnaire for socio-demographic characteristics and perceived associated factors of expired veterinary pharmaceuticals was adapted from literature (8, 17), and also for handling protocol of expired veterinary pharmaceuticals, was prepared based on the Ethiopian Medicines Waste Management and Disposal Directive (30). The qualitative data were collected through face-to-face interviews with key informants using a semi-structured interview guide that was developed from previously published literature in peer-reviewed journals (28, 29, 31–33).

### Data quality assurance

The data collected from all animal health facilities using the validated data collection forms were coded, checked for accuracy, consistency, omissions, and irregularities, and then prepared for analysis. The data collection tool for the study was pre-tested on 5% of the total sample size of the study, which was not included in the study. A data collector trained for 2 days on the data collection instruments and processes prior to data collection. The data collectors were supervised during the data collection process, and any inconsistencies were amended on time. For an in-depth interview, the interview guide was tested for its face and content validity was assured by the author team. It was prepared in the English language and translated into Amharic and then to English to check message consistency. The principal investigator spent an average of 30 min in-depth interviewing with the key informants. The principal investigator also used the reflexivity method to improve the rigor of the data collection, which enabled better probing, fewer assumptions, the avoidance of premature interpretation, and an emphasized sense of curiosity during the interview.

### Data management, analysis, and interpretation

In the quantitative part of the study, the magnitudes of expired veterinary pharmaceuticals were analyzed by Microsoft Office Excel version 10. Other quantitative parts of the study that assessed handling protocol and contributing factors were analyzed on the Statistical Package for Social Sciences (SPSS) version 25. The estimated money value (USD), unit pack, dosage form, types, and extents of expiration rates in each animal health facility were analyzed in Microsoft Excel. Veterinary pharmaceuticals were classified according to their anatomical therapeutic classes (ATC) using the classification of veterinary drugs adopted in the Ethiopian National Essential Veterinary Drug list (27).

The economic impact of expired veterinary pharmaceuticals was evaluated as per the Health Economic Evaluation Reporting Standards (34). The percentage of veterinary pharmaceutical wastage rate (%WR) was determined by calculating the percentage obtained by dividing the monetary value of wasted veterinary drugs by the total value of expired veterinary drugs received in the same year considering previously published peer-reviewed journals (28, 35, 36) using Equation 1.


(1) $$\begin{array}{l} \%WR=\\ \frac{\text{Value of wasted veterinary drug in}\;a\;\text{year}}{\text{Total value of veterinary drug received during the same year}}\;x\;100 \end{array}$$

If the rate of veterinary pharmaceutical expiration exceeds the permitted level of 2%, it indicates a higher rate of veterinary pharmaceutical expiration, and below 2% indicates a low expiration rate (37). The variables with higher mean values were considered the most associated factors for veterinary pharmaceutical expiration in animal health facilities. The categorical data were calculated as frequencies and percentages. The results were presented in tables, figures, and text form, depending on the nature of the variables under consideration.

For the qualitative study, face-to-face, in-depth interviews were conducted by the principal investigator. The audio recordings of in-depth interviews and discussions were transcribed by the investigator. Both the audio recordings and notes were translated into English. Subsequently, codes were assigned, and the issue was discussed. The codes were derived from the texts and categorized under the themes: current situation of expired veterinary pharmaceuticals, factors that contributed to expired veterinary pharmaceuticals, and potential strategies for mitigating the presence of expiration of such products. The number of key informants reached a saturation point in terms of information about the emerging themes. Finally, the text associated with each code was thoroughly examined, and the findings were presented through the use of quotations.

## Results

### The facility demographic profile

Of the 13 animal health facilities, 38.46% were veterinary clinics and 61.54% were private veterinary pharmacies. According to the survey, the stock of both kinds of veterinary clinics and pharmacies contains expired medications. When considering the year of establishment of these facilities, the highest number of respondents, eight (40%), stated that the facilities were established within a span of 5–10 years, whereas another eight (40%) reported that the facilities had been in existence for over 10 years. In this study, 14 (70%) of the respondents had additional funds allocated for the procurement of veterinary pharmaceuticals. None of the respondents had acquired veterinary pharmaceuticals near their expiration date in their facility. The details of the facility profile can be found in Table 1.

**Table 1:** Animal health facility demographic characteristics in the study area.

Study variables	Category	Frequency (*n* = 20)	Percentage (100%)
Animal health facility level	Veterinary pharmacies	8	61.54
	Veterinary clinics	5	38.46
Ownership	Private veterinary clinic	2	15.39
	Governmental veterinary clinic	3	23.07
	Private veterinary pharmacy	8	61.54
Year of established	<5 years	4	20.0
	5–10 years	8	40.0
	>10 years	8	40.0
Has the facility extra budget for drug procurement?	Yes	14	70.0
	No	6	30.0
Procure near expired veterinary drugs	Yes	0	0
	No	20	100.0

### The personnel demographic profile

The majority of the respondents (70%) were aged between 30 and 50 years. Of the total number of study participants, 85% were men and 15% were women. A significant portion of the participants 16 (80.0%) were married. In terms of profession, the study primarily consisted of veterinarians, accounting for the majority of respondents at 94.5%, followed by store managers, who accounted for 8 (40%). The details of socio-demographic characteristics can be found in Table 2.

**Table 2:** Socio-demographic characteristics of the respondents in the study area.

Background information	Category	Frequency (*n* = 20)	Percentage (100%)
Age	<30 years	3	15.0
	30–50 years	14	70.0
	>50 years	3	15.0
Sex	Male	17	85.0
	Female	3	15.0
Marital status	Married	16	80.0
	Widowed	0	0
	Single	4	20.0
	Divorced	0	0
Profession of drug store/clinic manager	Veterinarian	9	45.0
	Store manager	8	40.0
	Animal science expert	3	15.0
Year of experience	<5	5	25.0
	5–15	11	55.0
	>15	4	20.0
Educational level	Diploma	2	10.0
	Degree/DVM	17	85.0
	MSc	1	5.0

DVM, Doctor of Veterinary Pharmacy.

### The magnitude of expired veterinary pharmaceuticals in animal health facilities

A sum of 612 unit packs, comprising various anatomical therapeutic classifications (ATC) and dosage forms, with an estimated value of 69564.542 USD, expired within the animal health facility in and around Dessie town in the four fiscal years, spanning from 2019/2020 to 2021/2023 G.C. In the fourth fiscal year, the overall waste rate of expired veterinary pharmaceuticals in animal health facilities amounted to 7%.

A total of 13 animal health facilities were involved in the study; the majority, 11 (85%), of the facilities, received high expiration rates, whereas two (15% of them) received low expiration rates. The details of the magnitude of expired veterinary pharmaceuticals in terms of unit pack, expired cost in USD, purchased cost in USD, and expired rate were assessed in the respective animal health facilities (AHF) (Table 3).

**Table 3:** The magnitude of expired veterinary pharmaceuticals in four financial years.

Code	Expired unit pack	Expired cost in USD	Purchased cost in USD	Expire rate (%)
AHF-1	27	4841.663	44519.946	10.88
AHF-2	42	9244.594	104660.843	8.84
AHF-3	18	2648.958	45397.806	5.835
AHF-4	9	1612.029	98860.435	1.631
AHF-5	18	2942.863	77002.529	3.822
AHF-6	56	14412.336	99711.467	14.454
AHF-7	11	1894.1688	52682.693	3.595
AHF-8	23	4297.893	68374.805	6.286
AHF-9	134	6444.625	62283.994	10.347
AHF-10	167	6125.2449	64976.415	9.427
AHF-11	32	5301.464	86251.759	6.147
AHF-12	38	3286.200	177138.963	1.855
AHF-13	37	6512.503	85360.637	7.629
**Total**	**612**	**69564.542**	**1067222.292**	7%

1 US$ = 56.146 Birr, an average conversion rate has been used (December 2023).

### Magnitude of expired veterinary pharmaceuticals by animal health facility level

**Figure 2:**
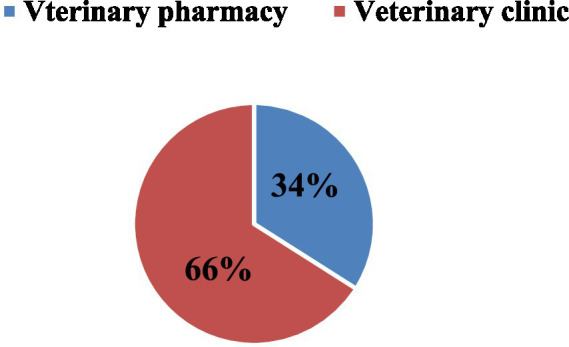
Magnitude of expired veterinary pharmaceuticals by unit packs at facility levels.

The monetary loss due to medicine expiration in private veterinary pharmacies amounted to 31,335.217 USD, while veterinary clinics experienced a loss of 38,229.329 USD. Overall, the expiration rate in veterinary pharmacies (*n* = 8) was 5%, whereas in veterinary clinics (*n* = 5) it was 10%. Comparatively, the proportion of expired veterinary pharmaceuticals seems to be less in veterinary pharmacies than in veterinary clinics (Figure 2 and Table 4).

**Table 4:** Level of animal health facility supply chain with the magnitude of expired veterinary drugs.

Indicator for the magnitude of expired veterinary drugs	Level of animal health facilities	
	Veterinary pharmacy (*N* = 8)	Veterinary clinic (*N* = 5)
Quantity of unit pack/percentage (*n* = 612)	209(34%)	403(66%)
Expired cost	31,335.217USD	38,229.329USD
Purchased cost	668,865.868USD	398356.428USD
Expiration rate (%)	**5%**	**10%**

The bold values in the table indicates the magnitude (%) of expired veterinary pharmaceuticals within animal health facility level.

According to the animal health facility level, it was estimated that 209 (34%) unit packs of veterinary pharmaceuticals expired in private veterinary pharmacies, while 403 (66%) unit packs of veterinary pharmaceuticals expired in veterinary clinics as depicted in Figure 2.

### Expired veterinary pharmaceuticals by anatomical therapeutic class in terms of monetary loss and units pack

According to the anatomical therapeutic class (ATC) classification, antibiotics accounted for the highest percentage of expired veterinary pharmaceuticals (14.8%), followed by antihelminthics (13.8%) in terms of monetary loss. The lack of financial records prevented the determination of the cost of vaccines, thus making it impossible to assess in monetary terms. Instead, it had to be elucidated based on unit packs.

Vaccines were also expired but that was donated, and the cost was unknown, so that could not be explained in terms of expired cost in birr but in unit pack only. This study revealed that approximately 131596.938 USD were lost due to medicine expiration in animal health facilities. The waste rate, based on the ATC classification, was found to be 14.4% (Table 5). The details of the expired veterinary pharmaceuticals from other anatomical therapeutic classes are described in Table 5.

**Table 5:** Expired veterinary pharmaceuticals by anatomical therapeutic class.

Anatomical therapeutic class	Purchased cost in USD	Expired cost in USD	Expire rate (%)
Antibiotics	794833.506	117734.113	14.8
Anthelminthic	88101.423	12137.306	13.8
Antiseptics and disinfectants	16293.236	1045.489	6.4
Vitamins	12380.935	667.902	5.4
Electrolyte	230.649	12.13	5.3
Overall wastage rate (%)	**911839.748**	**131596.938**	14.4

The bold values in the table indicates that the monetary loss of expired veterinary pharmaceuticals by Anatomical Classification.

Additionally, a total of 632 unit packs of veterinary pharmaceuticals were found to be expired in animal health facilities as per the anatomical therapeutic classification. Among these, antibiotics had the highest rate of drug expiration with 216 packs (34%), followed by anthelminthic drugs with 210 packs (33%), and vaccines with 193 packs (31%). The details of the remaining expired veterinary pharmaceuticals, categorized according to the anatomical therapeutic classification, can be found in Table 6.

**Table 6:** Anatomical therapeutic class of expired veterinary pharmaceuticals by quantity of unit packs.

Anatomical therapeutic class	Quantity of unit pack	Percent
Antibiotics	216	34
Anthelmintics	210	33
Vaccine	193	31
Antiseptics and disinfectants	7	1
Electrolyte	3	0.5
Vitamins	3	0.5

**Figure 3:**
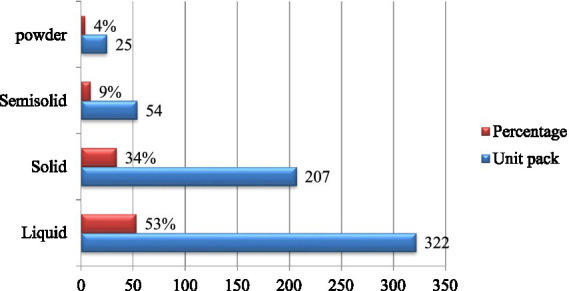
Percentage of Expired veterinary pharmaceutical dosage forms in terms of unit packs.

According to the dosage form analysis, a total of 322 unit packs (53%) of liquid veterinary pharmaceuticals were expired, whereas 207 unit packs (34%) of solid veterinary pharmaceuticals were also expired. The remaining expired dosage forms are detailed in Figure 3.

### Handling protocol and storage practices of expired veterinary pharmaceuticals

The handling protocol of the animal health facilities was determined by frequency and percentage. Among the 20 respondents, 60% had the necessary records and documentation for expired veterinary drugs in their facility. However, 40% of them did not have a separate and secure storage system for expired veterinary drugs, and 55% did not have procedures or protocols for disposing of these drugs. Approximately 35% of the respondents either did not reimburse or were at risk for the veterinary pharmaceuticals. Out of all the participants, 60% stored veterinary drugs for a long period without proper disposal, and 55% disposed of them poorly according to the recommended procedures at the facility. Furthermore, 65% of the respondents followed proper prescription and veterinary drug management practices to minimize the accumulation of expired pharmaceuticals in their facility (Table 7).

**Table 7:** Handling practice of expired veterinary drugs in animal health facilities.

Handling protocols	Measurement tool		
Protocols for expiring veterinary pharmaceuticals	Degree of agreement	Frequency (=20)	Percent
Necessary records for expired veterinary drugs	Strongly disagree	0	0
	Disagree	3	15.0
	Neutral	0	0
	Agree	12	60.0
	Strongly agree	5	25.0
Storage of expired veterinary drugs is separated from unexpired	Strongly disagree	2	10.0
	Disagree	8	40.0
	Neutral	0	0
	Agree	7	35.0
	Strongly agree	3	15.0
Procedure and programs for the disposal of expired veterinary drugs	Strongly disagree	4	20.0
	Disagree	11	55.0
	Neutral	1	5.0
	Agree	4	20.0
	Strongly agree	0	0
Expired veterinary drugs are reimbursed/lost money or at risk	Strongly disagree	1	5.0
	Disagree	7	35.0
	Neutral	2	10.0
	Agree	4	20.0
	Strongly agree	6	30.0
Expired veterinary drugs stored for a long time without disposal	Strongly disagree	1	5.0
	Disagree	1	5.0
	Neutral	1	5.0
	Agree	12	60.0
	Strongly agree	5	25.0
Dispose of concerned body recommendations	Strongly disagree	11	55.0
	Disagree	6	30.0
	Neutral	1	5.0
	Agree	2	10.0
	Strongly agree	0	0
Proper prescription of veterinary drugs	Strongly disagree	0	0
	Disagree	0	0
	Neutral	0	0
	Agree	7	35.0
	Strongly agree	13	65.0
Proper veterinary drug management to keep veterinary drugs from expiring	Strongly disagree	0	0
	Disagree	0	0
	Neutral	0	0
	Agree	7	35.0
	Strongly agree	13	65.0

### Factors associated with veterinary pharmaceutical expiration in the animal health facility

The evaluation of the animal health facility was conducted using a Likert scale, ranging from strongly disagreeing (*n* = 1) to strongly agreeing (*n* = 5). The questionnaires were distributed to the study population and received, resulting in a 100% response rate. The factors with the highest mean score indicated the most commonly perceived contributors to veterinary pharmaceutical expiration.

The mean score revealed that the lack of an information system and necessary software at the facilities (6 ± 0.1) was the most critical factor associated with veterinary pharmaceutical expiration. Additionally, poor store management (4.7 ± 0.5) and lack of strict accountability of the store manager (4.8 ± 0.4) were other factors associated with veterinary medicine expiration as reported by professionals working in animal health facilities (Table 8).

**Table 8:** Factors associated with veterinary drug expiration in animal health facilities.

Variables	SDA *n*(%)	DA *n*(%)	N *n*(%)	A *n*(%)	SA *n*(%)	Mean ± SD
Schedule for procurement	–	1(5)	–	14(70)	5(25)	4.2 ± 0.7
Selection and quantification during procurement depend on EDL (essential drug list)	6(30)	9(45)	1(5)	3(15)	1(5)	2.2 ± 1.2
Near-expiry veterinary drugs procuring	16(80)	2(10)	–	1(5)	1(5)	1.5 ± 1.1
Utilization of STG in the facility	–	4(20)	–	13(65)	3(15)	3.8 ± 1
Financing system for veterinary drug procurement	–	–	–	12(60)	8(40)	4.4 ± 0.5
Coordination with other supply chain staff	4(20)	7(35)	2(10)	3(15)	4(20)	2.8 ± 1.5
Accountability of veterinary store manager to reduce expired veterinary drugs	–	–	–	5(25)	15(75)	4.8 ± 0.4
Storage management	–	–	–	7(35)	13(65)	4.7 ± 0.5
Monthly physical count (physical count of items/drugs monthly)	–	–	–	16(80)	4(20)	4.2 ± 0.4
Use FEFO (first expired, first out) mechanisms	4(20)	2(10)	–	13(65)	5(25)	4 ± 0.8
Status of essential veterinary drug list at the facility	1(5)	11(55)	3(15)	2(10)	3(15)	4 ± 1.2
Status of SVTG at the facility	1(5)	4(20)	–	12(60)	3(15)	3.6 ± 1.1
Use information systems and necessary software at the facilities	20(100)	–	–	–	–	6 ± 0.1

*n*(%), frequency and percentage; SDA, strongly disagree; DA, disagree; N, neutral; A, agree; SA, strongly agree; and SD, standard deviation; EDL, essential drug list; SVTG, standard veterinary treatment guideline; FEFO, first expiry, first out.

### Qualitative information of the key informants

Key informants within the animal health facilities were interviewed to investigate the factors that lead to the expiration of veterinary drugs and to identify potential intervention strategies to mitigate the rate of veterinary pharmaceutical expiry in the animal health facility.

The interview consisted of a majority of participants (46%) who fell within the age range of 35 to 45 years. Additionally, the majority of participants (64%) were male. Among the study participants, 55% held a degree or DVM, and 64% had over 5 years of experience in their respective positions.

Furthermore, 46% of the study participants served as veterinary public health officers. It is important to note that all individual participants were selected from a total sample size of 20 individuals within the animal health facility in and around Dessie town as indicated in Table 9.

**Table 9:** Background information of the key informants.

Background Information	Category	Frequency (*N* = 10)	Percentage (100%)
Age	20–35	2	18.2%
	35–45	5	45.5%
	Above 45	3	27.3%
Sex	Male	7	63.6%
	Female	3	27.3%
Highest level of education	Diploma	-	-
	Degree/DVM	6	54.5%
	MSc Degree	4	36.4%
Work experience at position	1–5	3	27.3%
	Above 5	7	63.6%
Current position in the health facility	Veterinary Public Health Officer	5	45.5%
	Veterinary pharmacist	2	18.2%
	Director of clinic/pharmacy owner	3	27.3%

DVM, Doctor of Veterinary Medicine, MSc.

From the total interview, the following information was gathered about expired veterinary pharmaceuticals, which was categorized into three main themes: the current situation of expired veterinary drugs in the animal health facility, factors that contribute to the expiration rate of veterinary pharmaceuticals, and possible intervention mechanisms to reduce the extent of expired veterinary drugs. In order to maintain confidentiality, a unique code KI (Key-informant) was allocated to every key study participant, taking into account redundancy that determines the saturation point.

### Theme 1. Current situation of expired veterinary drugs

The majority of the interviewers answered the question “How do you assess the current situation of expired veterinary pharmaceuticals in your facility?” The expiry of veterinary pharmaceuticals is a common issue, particularly for drugs that are excessively purchased at a time and are more likely to expire than those drugs purchased with a purchasing schedule from a veterinary drug wholesaler and other shops. Respondent KI mentioned that:

*“In the facility, veterinary drugs are at risk because of their expiration; the facility has no separate stores for expired veterinary drugs and disposal schedules; and also, distributors are not collecting back the expired veterinary drugs.”* Additionally, the respondents also said, *“we are trying to separate expired veterinary drugs and dispose of them properly twice a year in the future.”*

One KI informant also highlighted the existing state of veterinary medications within the examined establishment: the absence of a proper procedure for segregating expired drugs from non-expired ones. Furthermore, the respondent said:

*“The expiry rate of veterinary pharmaceutical is evaluated twice a year, then expired drugs are separated from non-expired and stored separately by recording them in the record book and also those expired drugs are reported to the woreda Veterinary Health Office. There is also planned to reduce the amount of expired veterinary drugs by working hard with store managers and concerned bodies in the future, but currently, it is a challenging task to prevent expiration because of different challenges like lack of coordination with different stakeholders and staff.”*

### Theme 2. Factors that contributed to expired veterinary pharmaceuticals

For the question “What are the factors that contribute to expired veterinary drugs in your facility?” All respondents listed different factors responsible for the expiry of veterinary pharmaceuticals from their experiences. The majority of the respondents agreed on the lack of implementation of policy and guidelines. Moreover, poor store management systems at animal health facilities as factors that contributed to the expiry of veterinary pharmaceuticals at their respective facility as mentioned by the key informants.

One KI strongly highlighted that:

*“The primary factors contributing to the expiration of veterinary pharmaceuticals within the facility include the acquisition of donated drugs from governmental clinics and pharmacies, the procurement of multiple drugs simultaneously, and the* var*ying interest of customers in purchasing veterinary medications.”*

Additionally, two key informants mentioned that:

*“The most common factor responsible for expired veterinary pharmaceuticals in animal health facilities is improper storage; do not apply expiry prevention mechanisms like FIFO.”* Apart, *“poor inventory management and a lack of implementation of policies and guidelines were the main reasons for the high expiration rate of veterinary pharmaceuticals in the facility.”*

Several participants provided their responses to the inquiry regarding the impact of an expired veterinary drug on the service delivery by their facilities. They mentioned that:

*“Every possible measure was taken to enhance inventory management in order to minimize the occurrence of expired veterinary pharmaceuticals. In most cases, wholesalers and other veterinary drug stores were notified to refrain from distributing veterinary drugs that were nearing their expiration date within the market chain.”*

Furthermore, the key informants indicated that:

*“The economic impact on animal health facilities is significant when valuable veterinary drugs expire. Additionally, the lack of a secure and sufficient storage facility has led to environmental hazards. Moreover, this situation has placed an additional burden on staffs who work in the animal health facility, as they are responsible for the proper disposal of these drugs.”*

Additionally, Key informants mentioned that:

*“Method of disposing of expired veterinary pharmaceuticals involved burning and discarding them into the environment. However, these current disposal mechanisms pose potential risks for future health environments, and the general public health.”*

### Theme 3. Potential strategies for mitigating the prevalence of expired veterinary pharmaceuticals

The study participants mentioned several strategies for combating expired veterinary pharmaceuticals as potential mitigation strategies. Their recommendations were consistent and focused on improving veterinary pharmaceutical management practices and regulatory measures. The suggested strategies were categorized under sub-themes, which were detailed as follows:

#### Implement FIFO (first in, first out) mechanism

The key informants were mentioned that:

*“Proper use of the FIFO system is essential to prevent wastage due to expired medications. By ensuring that older stock is used before newer stock, the likelihood of drugs expiring before use is minimized.”*

#### Avoid procurement and distribution of near-expiry veterinary pharmaceuticals

Regarding the prevention of procurement and distribution of near-expiry drugs, the key informants emphasized the following points:

*“Prevent the procurement and distribution of veterinary drugs with near-expiry dates to reduce the risk of them expiring before they are used. This can be achieved through better coordination with suppliers and improved monitoring of stock levels.”*

#### Strengthen coordination and inventory management

Additionally, the study participants said that:

*“Enhance coordination between veterinary clinics and pharmacy professionals and establish robust inventory management systems at veterinary pharmaceutical stores. Additionally, effective inventory management can help prevent the accumulation of expired drugs and ensure the timely use of available stock.”*

#### Ensure qualified personnel

Regarding qualification, the key informants cited that:

*“Avoid placing non-certified individuals in the position of veterinary pharmaceutical store manager. Proper qualifications and training for personnel are critical for effective management of pharmaceutical inventory.”*

#### Develop policies and regulations

For the future roadmap of reducing the presence of expired veterinary pharmaceuticals in animal health facilities, the study participants recommended the following actions:

*“Develop, and enforce drug policies and regulations that govern the management of veterinary drugs, including those related to expiration. These policies should increase accountability among facility administrators and employees involved in drug management.”*

#### Facilitate redistribution of near-expiry veterinary pharmaceuticals

The study participants also mentioned that:

*“Develop policies and regulations to support the redistribution of near-expiry and surplus veterinary pharmaceuticals. Implement electronic reporting systems to facilitate information exchange between animal health facilities, enabling them to accept and donate such drugs efficiently.”*

## Discussion

Veterinary pharmaceuticals play a vital role in preventing disease and improving the productivity of livestock. These medications are used to prevent, control, and treat various diseases, thereby ensuring the wellbeing of animals (38). This, in turn, has a direct impact on food security, public health, and the economic stability of communities that rely on livestock for their livelihood (39). Nevertheless, the worldwide concern lies in the presence of expired drugs within the supply chain, which can pose risks to public health (40, 41). In this study, attempts were made to evaluate the magnitude, financial impact, and factors associated with expired veterinary pharmaceuticals in animal health centers in and around Dessie Town, South Wollo, Ethiopia.

### The magnitude of expired veterinary pharmaceuticals data by facility type

The assessment of the quantity of expired veterinary pharmaceuticals in animal health facilities is of utmost importance in terms of its economic implications and public health concerns (40). As per the results of the current study, of the 13 animal health facilities, 61.54% were veterinary pharmacies, while 38.46% were veterinary clinics. It was observed that both types of establishments had expired veterinary pharmaceuticals on their facilities. This could potentially suggest a lack of effective collaboration among animal health experts employed at the facility, the governing body responsible for veterinary supplies, the agency overseeing drug regulations, and other pertinent parties involved (41). According to research conducted in Uganda, 55% of participants obtained their veterinary medications from wholesalers; the Wakiso district also reported comparable results. Antimicrobials were also acquired from these sources for animals (28).

Additionally, a sum of 612 unit packs, comprising various anatomical therapeutic classifications (ATC) and dosage forms, with an estimated value of 69564.542 US Dollars, was expired within the animal health facility in and around Dessie town in the four fiscal years, spanning from 2019/2020 to 2021/2023. In the fourth fiscal year, the overall waste rate of expired veterinary drugs in animal health facilities amounted to 7%. Therefore, the current investigation has indicated that the expiration rate of veterinary pharmaceuticals in animal health facilities exceeded the permitted national expiration rate of 2% (37). The findings of study, which also showed that comparable results were seen in Uganda, approximately 66% of veterinary medications expired in clinics, and 34% in private pharmacies underline the administrative and financial costs related to drug expiration (8).

The study found that private veterinary pharmacies experienced a monetary loss of 31,335.217 USD due to pharmaceutical expiration, while veterinary clinics experienced a loss of 38,229.329 USD. The findings suggest that the need for effective store management and dispensing practices and veterinary professionals employed at the facility must implement effective store management techniques, along with enhanced inventory management and proficient dispensing practices such as FIFO, FEFO, LIFO, and LEFO (18). One of the issues of animal health supply chain management in underdeveloped nations, particularly Ethiopia, is veterinary pharmaceutical expiration. This was due to a lack of reliable data on the detailed underlying variables related to expired veterinary drugs in animal health facilities. Furthermore, based on the anatomical therapeutic class (ATC) classification, antibiotics constituted the largest proportion of expired veterinary pharmaceuticals (14.8%), followed by anthelminthic drugs (13.8%) in terms of financial loss. The findings of this research indicated that approximately 131596.938 USD was wasted as a result of medication expiration in animal health facilities. The waste rate, as determined by the ATC classification, was identified as 14.4%.

The study revealed that antibiotics had the highest rate of drug expiration, accounting for 216 packs (34%), followed by anthelmintics with 210 packs (33%), and vaccines with 193 packs (31%). This aligns with findings from Central Uganda, where antibiotics were also the most frequently expired drugs in human healthcare supply outlets, particularly injectable veterinary antibiotics, with 82.8% of respondents (24/29) reporting frequent expirations (8). This highlights a significant issue with the management and storage of these critical medications. The high rate of expired veterinary antibiotics in animal health facilities is a significant concern, especially regarding antibiotic resistance. When expired antibiotics are not properly disposed of, they can contribute to critical issues of antibiotic resistance development (42, 43). The development of antibiotic resistance can be greatly aided by the improper enforcement of veterinary pharmaceutical regulations for the appropriate disposal of veterinary medications that have expired, especially antibiotics in the environment. The situation in Ethiopia highlights this concern, where a report indicated that 75.5% of multidrug resistance (MDR) was observed in bacteria isolated from dairy farm sewage samples (44). As per the dosage form, 322 unit packs (53%) of liquid drugs were expired, whereas 207 unit packs (34%) of solid drugs were expired in the current animal health facilities. This may suggest a demanding need for immediate attention to be given to animal health facilities to effectively allocate the health budget for the distribution of veterinary pharmaceuticals (38).

Country level, veterinary medicines expiration is a universal pharmaceutical problem. For instance, a study conducted in Central Uganda showed that the total value of expired products for all program categories was valued at <200 million Ugandan shillings ($55,000) at the time of the research (8). This indicates that animal health facilities are under a large economic burden due to financial loss as a result of expired veterinary medicines; therefore, animal health professionals should improve the supply chain management and inventory management of facilities and work on the implementation of policies and guidelines.

### Handling protocol and storage practice of expired veterinary pharmaceuticals

In the current study, the handling protocol and storage practice of expired veterinary pharmaceuticals in the facilities were assessed based on a 5-point Likert scale that ranged from strongly disagree (*n* = 1) to strongly agree (*n* = 5). The study revealed that the majority (55% of the respondents) lacked disposal procedures or programs, and 60% of them were stored for an extended period of time before being returned. The study was inconsistent with the study conducted in Uganda, which showed high awareness of the environmental impacts of poor antimicrobial disposal (8). Another study conducted by Vatovec et al. (24) also supported this study, which indicates about veterinary professionals reported expired veterinary drugs stored without disposal programs. It indicates that a large volume of drugs go unused annually and that only a portion of leftover veterinary medications are returned to take-back programs where they can be appropriately disposed of, and expired veterinary drugs were not reimbursed or put at risk in the facility.

This indicates several unwanted veterinary medicines expire at a certain time. When expired pharmaceuticals are improperly stored and disposed of, they can lead to contamination and a wide range of toxicities in humans and animals (12, 45). Additionally, expenditure of useful veterinary drugs due to expire can cause economic implications. The study conducted by Kadam et al. (33) showed that habits of veterinary medicine disposal depend on socioeconomic culture as well as regulatory guidelines, and norms that prevail in the country. Hence, it is authoritative to establish government-operated support and disposal systems that are both cost-effective and widely accepted.

In the present study, nearly half of the animal health facilities visited did not continuously document expired veterinary drugs between FY 2019 and FY 2023. The reason given was the increased workload of the professionals. This indicates that a lot of veterinary medicines are expired but not reported in animal health facilities due to poor record management. Literature explores future trends in archiving veterinary pharmaceutical documentation and proposes necessary standards and mechanisms for the task as well as the information model of submission and archival information packages for this domain (46). Hence, animal health facilities should incorporate adequate documentation systems and record management.

### Contributing factors for veterinary pharmaceutical expiration

The presence of pharmaceuticals in the environment is a globally acknowledged issue that is of concern on a daily basis. Environmental contaminants in the form of drugs have been identified in water and soil systems, thereby posing potential risks to both humans and wildlife (47). Identifying the primary factors that contribute to the expiration of veterinary pharmaceuticals is crucial in order to effectively prevent the disposal of expired drugs in the environment, and the economical wastage of the health budget. In this study, the lack of an information system and necessary software at the facilities was the most critical factor associated with veterinary medicine expiration. Additionally, the lack of strict accountability of the store manager and poor store management were the most critical factors associated with veterinary medicine expiration were another factors that fired the path of veterinary medicines expiration. Therefore, there should be strict control and regulation of animal health facilities to implement policies and guidelines as well as facilitate proper store management.

The presence of poor inventory management and administrative systems in the facility was also a factor contributing to veterinary drug expiration, as per the present findings. Similarly, a study performed in Uganda by Manyi-Loh et al. (40) showed that poor inventory management of veterinary medicines was one of the major contributing factors to the expiration of veterinary medicines. Inadequate inventory control in animal health facilities results in a high rate of expired medications, elevated storage expenses, and theft. This leads to the underutilization of consumption data and the involvement of experts in prescription and drug forecasting. In this study, a qualitative study was also performed to understand the current situation of expired veterinary drugs in animal health facilities, factors that contribute to the expiration rate of veterinary drugs, and possible intervention mechanisms to reduce the extent of expired veterinary drugs. Due to a lack of separate storage, irregular disposal schedules, and distributors who fail to retrieve expired veterinary medications, the situation involving these products at animal health facilities requires immediate attention. Although there are initiatives to scale back, there are a number of obstacles to overcome, such as poor planning and insufficient storage space (48). The primary contributing factors to veterinary pharmaceutical issues include improper storage practices, failure to implement expiry prevention mechanisms, poor inventory management, and lack of policy implementation, leading to income loss and unfavorable consequences (49).

### Potential strategies for mitigating the expiration of veterinary pharmaceuticals

This study identified several critical strategies for reducing the prevalence of expired veterinary pharmaceuticals. Mitigation parameters such as robust coordination, effective inventory management, clear policy formulation, careful purchasing to avoid near-expiry products, improved dispensing procedures, and using electronic-based reporting for data sharing reflect a comprehensive approach to tackling this issue. By facilitating coordination and information exchange, animal health facilities can prevent overstocking and anticipate expiration dates more effectively. The parallel with human pharmaceutical supply chain management in Ethiopia underscores the universal relevance of these interventions (29). Implementing similar strategies across both veterinary and human pharmaceutical systems could help streamline inventory, reduce waste, and improve the availability of quality pharmaceuticals in healthcare settings.

The findings of the study on the importance of hiring qualified, certified professionals as veterinary pharmaceutical store managers highlight a critical step for ensuring proper inventory management and reducing expired veterinary pharmaceuticals. Employing well-trained personnel with accredited qualifications can significantly improve inventory tracking, storage conditions, and timely usage of drugs, leading to fewer expirations and waste. A report from South Africa emphasized the significance of licensing individuals involved in compounding, dispensing, or manufacturing medicines. This licensing process excludes veterinarians due to a ruling by the Constitutional Court (50). The decision undermines the critical role that proper licensing plays in ensuring the effective and safe use of medications. This regulation is especially relevant in maintaining the standards for medicine quality, safety, and availability, with licensed professionals held accountable to established protocols and regulations (50).

A similar report identified from Uganda indicates that implementing inventory tracking technology is a valuable policy recommendation for improving veterinary pharmaceutical management (8). For a sustainable future framework, the recommendation to develop and enforce clear drug policies and regulations specifically tailored to veterinary pharmaceutical management is key. Such policies would not only provide guidance on handling and monitoring expiration dates but also establish accountability among facility administrators and employees responsible for veterinary pharmaceutical management.

By encouraging information sharing, efficient inventory control, and a reduction in expired medication, communities of practice can greatly assist veterinary professionals in South Wollo. They can also improve policymaking and practical implementation. Communities of practice (CoPs) bring together individuals with diverse skill sets who actively engage in various activities, such as exchanging knowledge, offering tips, and sharing innovative ideas. This collaborative approach is crucial for identifying and disseminating valuable resources that can enhance the utilization of veterinary-related infrastructure by educators in the field (49). Depending on the current finding and from the existing evidence, the study concluded that better inventory management practice, good disposal protocol, and the implementation of the policy framework and guidelines play a critical role in the reduction in veterinary pharmaceutical expiration. Additionally, establishing communities of practice could be a viable solution for the existence of veterinary pharmaceutical expirations.

### Limitations of the study

The study has limitations that impacted its coverage and generalizability. Financial limitations confined the research to veterinary clinics and pharmacies within the Dessie area, limiting the applicability of the study to a broader regional or national context. Additionally, the practice of mixing veterinary pharmaceuticals in various formulations created challenges in accurately quantifying expired drugs as the cost-based approach used to estimate expired stock did not fully capture the magnitude of expiry issues. Records on expired veterinary pharmaceuticals were primarily due to data damage during the 2022 Ethiopian conflict, which further limited access to historical information. The small sample size, with participants drawn only from a limited selection of veterinary clinics and private pharmacies, contributed to a less comprehensive investigation. Another major weakness was the absence of quantitative data on the environmental impact of expired veterinary pharmaceuticals. Future studies should incorporate detailed environmental data on a comprehensive understanding of the impact of expired veterinary pharmaceuticals.

### Strengths of the study

Despite its limitations, the study was robust in its methodology, utilizing both quantitative and qualitative approaches to provide a thorough understanding of the issues. This mixed-methods approach allowed for addressing observed gaps effectively. By focusing on the Dessie area, the study offered in-depth, localized insights specific to the operational challenges faced by veterinary clinics and pharmacies. Additionally, it sheds light on financial and operational challenges related to managing expired veterinary pharmaceuticals, contributing valuable insights toward improved management practices in veterinary pharmaceutical sectors.

## Conclusion and outlooks

The study reveals a significant issue with the expired veterinary pharmaceuticals, which accounted for a 7% overall expiration rate, leading to a financial loss of 69564.542 USD in the study area. Antibiotics were particularly impacted, representing 14.8% of the total value lost. Approximately 53% of liquid dosage forms were expired. The handling and disposal practices for expired veterinary pharmaceuticals were found to be inadequate, with a lack of standardized storage systems and proper disposal procedures. The study identifies several contributing factors to the high expiration rates, including improper dispensing practices, simultaneous procurement of multiple drugs, inconsistent customer demand for veterinary medications, poor inventory management, and insufficient implementation of relevant policies and guidelines. Hence, collaboration with concerned bodies such as the Ethiopian Agricultural Authority, pharmaceutical supply chain agencies, researchers, and healthcare veterinary professionals is critical for improving veterinary pharmaceutical purchasing strategies, enhancing storage and disposal systems, implementing inventory management systems, and policy and guideline enforcement. Such efforts will be vital for combating the presence of expired veterinary pharmaceuticals and monetary loss.

## Data Availability

The original contributions presented in the study are included in the article/Supplementary material, further inquiries can be directed to the corresponding author.
