# Supplementary material for: Assessment of the magnitude, economic impact, and factors associated with expired veterinary pharmaceuticals in animal health facilities in South Wollo, Ethiopia
Source: Front Vet Sci. 2025 Jan 7;11:1390891. doi: 10.3389/fvets.2024.1390891 (PMC11747527; doi:10.3389/fvets.2024.1390891)
Supplement: Supplementary file 2 [file Table_2.DOCX]

**Supplementary File 2. Checklist for evaluation of animal health facilities regarding handling of expired veterinary drugs.**

| S/N | **Protocols for expired veterinary drug** | Degree of agreement | | | | |
| --- | --- | --- | --- | --- | --- | --- |
|  |  | 1 | 2 | 3 | 4 | 5 |
| 1 | Necessary records for expired Veterinary drugs |  |  |  |  |  |
| 2 | Storage of expired Veterinary drug are separated from unexpired |  |  |  |  |  |
| 3 | Procedure and programs for disposal of expired Veterinary drugs |  |  |  |  |  |
| 4 | Expired veterinary dugs are reimbursed/lost money or at risk |  |  |  |  |  |
| 5 | Expired veterinary drugs stored for long time without disposal |  |  |  |  |  |
| 6 | Dispose as concerned body recommendations |  |  |  |  |  |
| 7 | Proper prescription of Veterinary drugs |  |  |  |  |  |
| 8 | Proper veterinary drug management to keep Veterinary drugs from expire |  |  |  |  |  |

**Note:** 1 (Strongly Disagree), 2 (Disagree), 3 (Neutral), 4 (Agree), 5 (Strongly Agree)
