# Supplementary material for: Assessment of the magnitude, economic impact, and factors associated with expired veterinary pharmaceuticals in animal health facilities in South Wollo, Ethiopia
Source: Front Vet Sci. 2025 Jan 7;11:1390891. doi: 10.3389/fvets.2024.1390891 (PMC11747527; doi:10.3389/fvets.2024.1390891)
Supplement: Supplementary file 3 [file Table_3.DOCX]

**Supplementary File 3.** Guide for evaluation of animal health facilities regarding factors associated to expired Veterinary drugs

| **Variables** |
| --- |
| **Veterinary pharmaceutical Inventory management** |
| Schedule for Procurement |
| Selection and quantification during procurement is depend on EDL (Essential Drug List) |
| Near expiry Veterinary drugs procuring |
| Utilization of STG (Short Term Goals) in the facility |
| **Administrative system** |
| Financing system for Veterinary drug procurement |
| Coordination with other supply chain stuff (e.g., Vet. Colleges/universities) |
| **Store** |
| Accountability of Veterinary store manager to reduce expired veterinary drugs |
| Storage management |
| Monthly physical count (physical count of items/drugs monthly) |
| Use FEFO (First Expired, First Out) mechanisms |
| **Implementation of policy and guideline** |
| Status of essential Veterinary drug list at the facility |
| Status of STG (Short Term Goals) at the facility |
| Use Information system and Necessary software at the facilities |

**Note:** SA; Strongly Disagree (1), A; Disagree (2), N; Neutral (3), DA; Agree (4), SDA; Strongly Agree (5)
